# Association between triglyceride-glucose index and 6-month readmission in patients with heart failure: a cohort study

**DOI:** 10.3389/fcvm.2025.1547755

**Published:** 2025-07-29

**Authors:** Huibo Li, Yujie Jiang, Dong Zheng, Guixiong Lin, Yanling Feng, Yufeng Zhuo, Peng Zhang

**Affiliations:** ^1^ Department of Cardiology, Panyu Maternal and Child Care Service Centre of Guangzhou, Guangzhou, Guangdong, China; ^2^ Guangzhou Panyu District Maternal and Child Care Family Planning Service Center, Guangdong, China

**Keywords:** triglyceride-glucose index, heart failure, insulin resistance, BMI, cohort study

## Abstract

**Background:**

Insulin resistance (IR) significantly impacts outcomes in heart failure (HF) patients, by the triglyceride-glucose (TyG) index serving as an effective marker of metabolic dysfunction. However, its association with HF readmission risk is still uncertain.

**Methods:**

This study analyzed 901 HF patients using multivariable logistic regression and restricted cubic splines to evaluate the relationship between the TyG index and 6-month readmission risk.

**Results:**

Patients in the lowest TyG quartile (Q1) had a significantly higher risk of 6-month readmission across models: Model 1 [odds ratio (OR) 1.72, 95% confidence interval [(CI) 1.16–2.53; *p* = 0.007], Model 2 (OR 1.83, 95% CI 1.23–2.72; *p* = 0.003), and Model 3 (OR 1.71, 95% CI 1.12–2.61; *p* = 0.012). A nonlinear relationship between the TyG index and the 6-month readmission risk was observed (p for nonlinearity = 0.046). Furthermore, significant interactions were detected between the TyG index and body mass index (BMI) subgroups (*p* for interaction = 0.002). Including the TyG index modestly improved predictive performance, particularly in abnormal BMI patients.

**Conclusion:**

A nonlinear relationship between the TyG index and 6-month readmission risk in HF patients. Subgroup analyses revealed that a lower TyG index was significantly connected to a higher readmission risk, especially in patients with abnormal BMI. Although the TyG index improved predictive performance, its overall discriminative ability remained modest, demonstrating greater utility in populations with abnormal BMI.

## Introduction

Heart failure (HF) has emerged as a significant global public health concern since the beginning of the 21st century (1). Its increasing prevalence is closely tied to the aging population and advancements in treatment strategies (2). According to the Chinese HF guidelines, the prevalence of HF has surged six-fold over the past decade, and the mortality rate for patients with severe HF approaches 50% (3). Despite improved survival rates post-diagnosis, more than 20% of patients with HF face readmission within 30 d and approximately 50% within 6 months (4). This highlights the critical need for reliable indicators to identify high-risk individuals and enable the implementation of early preventive strategies.

Numerous clinical studies have highlighted the role of metabolic disturbances, particularly insulin resistance (IR), in the development of cardiac disease and the reduced effectiveness of pharmacological treatments (5). The triglyceride-glucose (TyG) index, a new and straightforward measure of IR, has garnered attention owing to its simplicity and emerging association with cardiovascular disease risk, including HF (6).

Although previous research has explored the relationship between the TyG index and HF, there has been limited exploration of its correlation with patient readmissions. This study aimed to investigate the potential association between the TyG index and the likelihood of 6-month readmission in patients with HF.

## Methods

### Study design and data source

**Figure 1:**
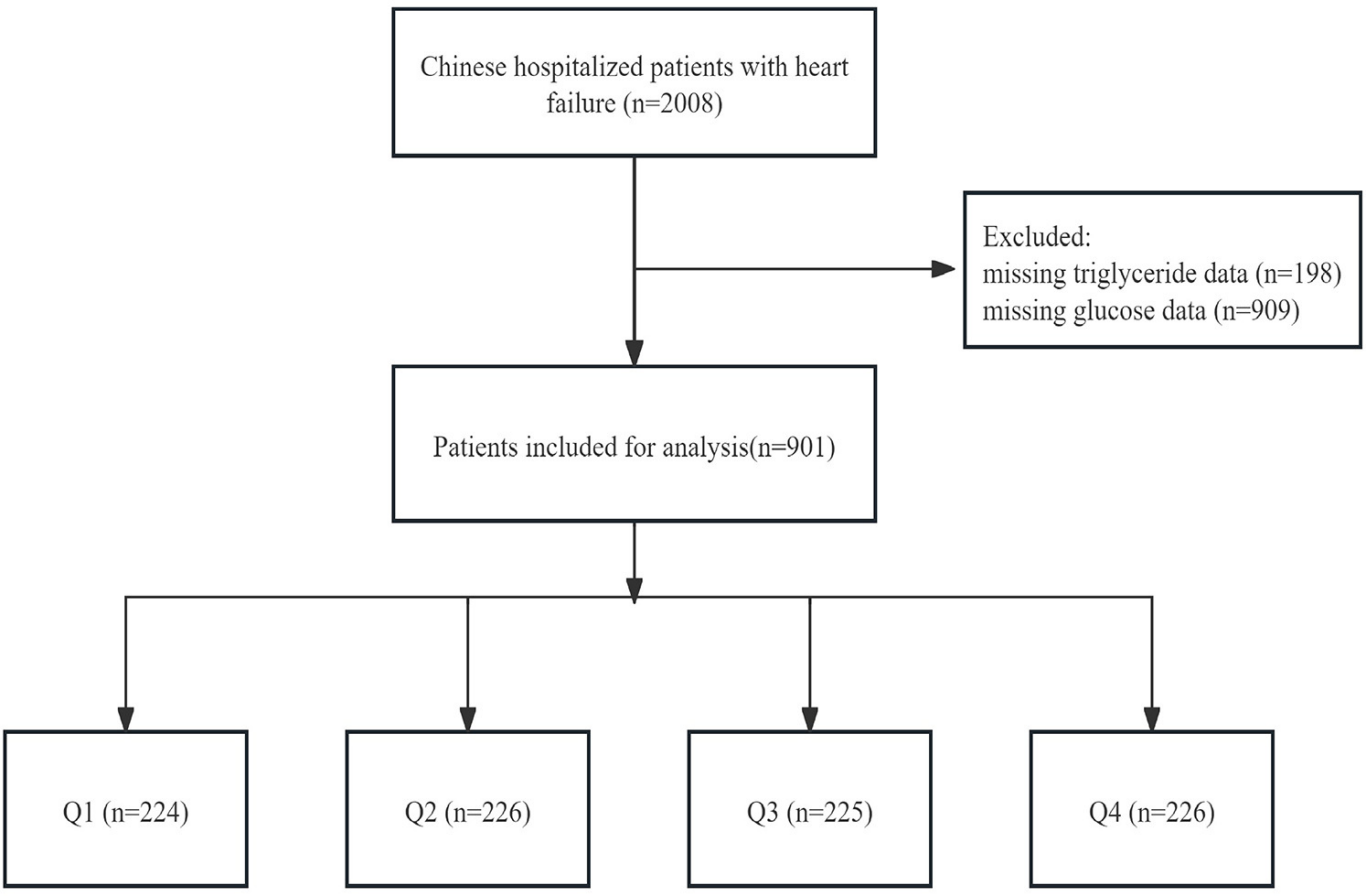
The flowchart of patients’ inclusion and exclusion.

This analysis was a retrospective study conducted at a single centre, using data sourced from the PhysioNet platform spanning the period from December 2016 to June 2019 (https://doi.org/10.13026/8a9e-w734) (7). This dataset included consultation records and follow-up information of 2,008 patients with HF admitted to the Zigong Fourth People's Hospital in Sichuan, China. HF was defined according to criteria set by the European Society of Cardiology (8). The study followed the STROBE guidelines (Strengthening the Reporting of Observational Studies in Epidemiology) and was approved by the Zigong Fourth People's Hospital Ethics Committee (approval number: 2020-010) in accordance with the Declaration of Helsinki. Due to the retrospective design of the study, informed consent was waived. We included 901 patients in the primary analysis after excluding those without recorded glucose and triglyceride levels (Figure 1).

### Independent variable, outcome

The TyG index, considered to be the primary exposure variable, was determined using the equation: ln [TG (mg/dl) × fasting blood glucose (mg/dl)/2] (9). The primary outcome of interest was the assessment of the risk of 6-month readmission for HF.

### Potential covariates

Clinical characteristics and medical histories were collected upon admission, including demographics and vital sign parameters like age, gender, pulse rate, mean arterial pressure (MAP), and body mass index (BMI) was classified into two categories: Normal (18.5–24.0 kg/m^2^) and Abnormal (<18.5 kg/m^2^ or ≥24.0 kg/m^2^), which is described in the Guidelines for Prevention and Control of Overweight and Obesity in Chinese Adults (10). The New York Heart Association (NYHA) classification was classified into two categories: Ⅱ and Ⅲ/Ⅳ (11), and HF along with comorbidities, such as congestive heart failure (CHF), peripheral artery disease (PAD), cerebrovascular disease, chronic obstructive pulmonary disease (COPD), diabetes, and chronic kidney disease (CKD). Laboratory results within 24 h of admission included estimated glomerular filtration rate (eGFR), white blood cell, hemoglobin, platelet, calcium, potassium, chloride, sodium, lactate, brain natriuretic peptide (BNP), albumin, cholesterol, low-density lipoprotein cholesterol (LDL-C), triglycerides, and high-density lipoprotein cholesterol (HDL-C). The medications administered during hospitalization, such as vasodilators, beta-blockers, renin-angiotensin-aldosterone system (RAS) inhibitors, inotropes, statins, and diuretics, were documented.

### Statistical analysis

Continuous variables are expressed as the mean and standard deviation (SD), while categorical variables are summarized as frequencies and percentages. Differences among categorical variables were evaluated using Chi-square tests, and analysis of variance (ANOVA) was applied to compare continuous variables across quartiles of the TyG index to describe demographic characteristics.

Multivariate logistic regression was conducted to determine the odds ratios (ORs) and 95% confidence intervals (CIs) to examine the relationship between the TyG index and 6-month readmission. Three models were developed: Model 1 was unadjusted, serving as a baseline; Model 2 was adjusted for demographic and clinical variables such as age, gender, BMI, NYHA, CHF, PAD, cerebrovascular disease, COPD, diabetes, and CKD; and Model 3 included all adjustments from Model 2 with additional adjustments for laboratory values and medications, including eGFR, white blood cell, hemoglobin, platelet, calcium, BNP, albumin, lactate, cholesterol, and pharmacological treatments including vasodilators, beta-blockers, RAS inhibitors, inotropes and statins. Covariate adjustments were based on clinical considerations, a 10% change in matched ORs, and *p*-values < 0.05, as observed in the univariate analyses (12). To investigate the link between the TyG index and 6-month readmission, smooth curve fitting was performed after adjustments in Model 3.

Following the adjustments made in Model 3, we explored the stability of the association across populations through interaction and subgroup analyses based on BMI (normal vs. abnormal) and diabetes status (no vs. yes), with the results displayed in a forest plot. As an additional exploratory analysis, we examined the relationship between the TyG index and 6-month readmission in normal and abnormal BMI groups with adjustments made according to model 3. The receiver operating characteristic (ROC) curves were utilized to assess whether incorporating the TyG index, as a categorical variable, improved the predictive performance of a baseline risk model. The model was adjusted for variables in the overall patient population, including diabetes, estimated glomerular filtration rate (eGFR), calcium, albumin, cholesterol, and beta-blockers. Additionally, it was adjusted for variables in patients with abnormal BMI, which included diabetes, calcium, albumin, hemoglobin, and statins. The area under the curve (AUC) for the models was compared utilizing DeLong's test. Furthermore, additional performance metrics, such as net reclassification improvement (NRI) and integrated discrimination improvement (IDI), were calculated to quantify the incremental predictive value of the TyG index. Statistical analyses were conducted using the Free Statistics software (version 1.9.2) and R (version 4.2.1) (http://www.R-project.org, R Foundation). Statistical significance was defined as a two-sided *p*-value of less than 0.05.

## Results

### Initial demographic and clinical features of patients with HF

There were 901 patients with HF extracted from the database. The baseline clinical and laboratory characteristics of these patients were stratiﬁed by quartiles of the TyG index, as presented in Supplementary Table S1. Among them, 76.0% were older than 70 years and 55.5% were female. The average TyG index was 8.6 ± 0.7. Given that the second quartile showed the lowest incidence of 6-month readmission (67/901 patients), we used the second quartile as the reference. As the TyG index increased across the quartiles, the proportions of diabetes and the use of vasodilators, beta-blockers and statins increased.

### Associations between TyG index and 6-month readmission risk

**Figure 2:**
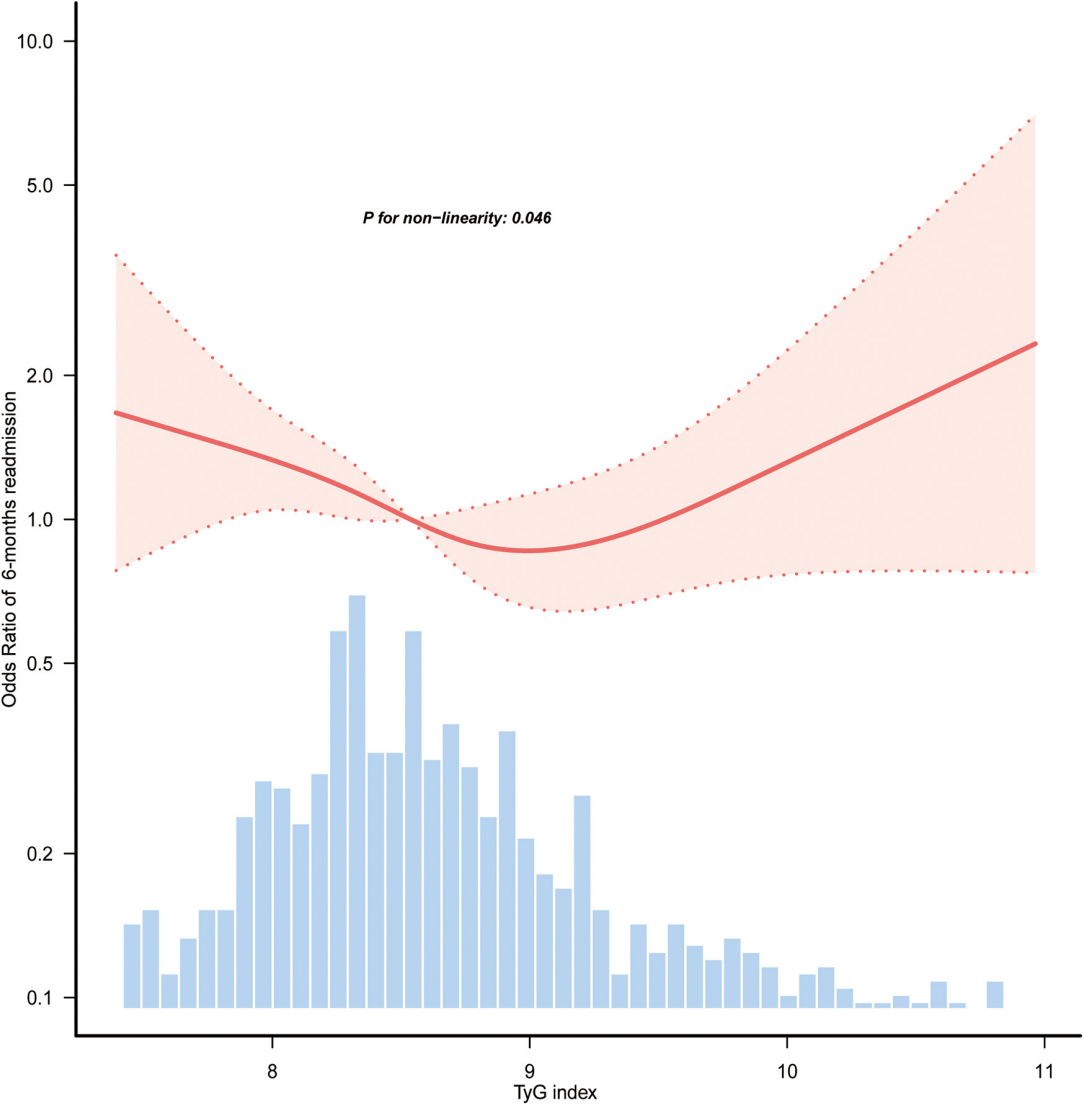
Restricted cubic spline curves of the association between TyG index and 6-month readmission of HF. Covariables were adjusted as in model 3 (Table 1). Only 98.5% of the data is shown. TyG, triglyceride-glucose; HF, heart failure.

Table 1 presents the findings from the multivariate logistic regression analysis assessing the relationship between the TyG index and the likelihood of 6-month readmission. Stratifying the TyG index into categories, it was observed that patients in the Q1 category exhibited a significantly greater risk of readmission compared to those in the Q2 category. The ORs were 1.72 (95% CI: 1.16–2.53; *p* = 0.007), 1.83 (95% CI: 1.23–2.72; *p* = 0.003), and 1.71 (95% CI: 1.12–2.61; *p* = 0.012) across the different models. Figure 2 illustrates a nonlinear link between TyG index levels and the risk of 6-month readmission for patients with HF (*p* for nonlinearity = 0.046).

**Table 1:** Relationship between TyG index and 6-month readmission of HF in different models.

Variable	Event%	Model 1	*P* value	Model 2	*P* value	Model 3	*P* value
TyG quartiles		OR (95% CI)		OR (95% CI)		OR (95% CI)	
Q1	94 (42.0)	1.72 (1.16∼2.53)	0.007*	1.83 (1.23∼2.72)	0.003*	1.71 (1.12∼2.61)	0.012*
Q2	67 (29.6)	1 (Reference)		1 (Reference)		1 (Reference)	
Q3	74 (32.9)	1.16 (0.78∼1.73)	0.458	1.17 (0.78∼1.77)	0.443	1.21 (0.79∼1.85)	0.393
Q4	75 (33.2)	1.18 (0.79∼1.75)	0.418	1.03 (0.67∼1.58)	0.888	1.00 (0.63∼1.60)	0.994

Model 1: not adjusted. Model 2: Model 1 + age, gender, BMI, NYHA, CHF, PAD, cerebrovascular disease, COPD, diabetes and CKD. Model 3: Model 2 + eGFR, white blood cell, hemoglobin, platelet, calcium, BNP, albumin, lactate, cholesterol, vasodilators, beta-blockers, RAS inhibitors, inotropes and statins. TyG, triglyceride-glucose; HF, heart failure; BMI, body mass index; NYHA, New York Heart Association; eGFR, estimated glomerular filtration rate; BNP, brain natriuretic peptide; RAS inhibitors, renin-angiotensin-aldosterone system inhibitors. OR, Odds Ratio.

*P < 0.05.

### Subgroup analyses

**Figure 3:**
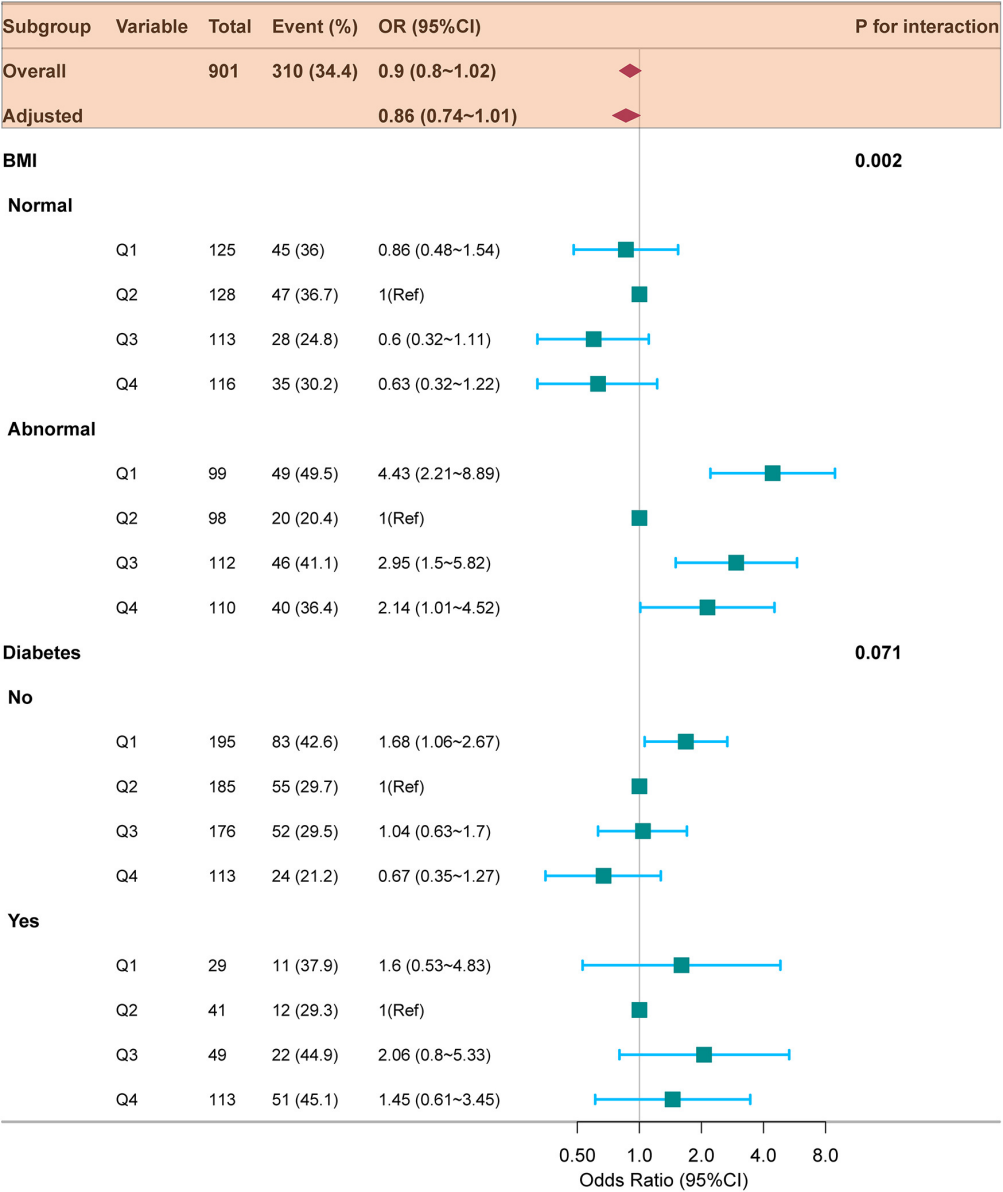
Forest plot for subgroup analysis of the relationship between TyG index quartiles and 6-month readmission of HF. ORs (95% CIs) were derived from multiple logistic regression models. Covariables were adjusted as in model 3 (Table 2). TyG, triglyceride-glucose; HF, heart failure; BMI, body mass index; OR, odds ratio; CI, confidence interval.

Subgroup and interaction analyses were conducted to assess the stability of the relationship between TyG index and the likelihood of readmission within 6-month. According to Figure 3, significant interactions were identified between TyG index and 6-month readmission risk in the BMI subgroup (*p* for interaction = 0.002), whereas no significant interaction was detected in the diabetes subgroup (*p* for interaction = 0.071). In patients with abnormal BMI, those with the lowest TyG index (Q1) were linked to a higher risk of readmission (OR = 2.76, 95% CI: 1.53–4.99; *p* = 0.001). Table 2 aligns with the forest plot results, in the abnormal BMI patients, the lowest TyG (Q1) was highly associated with increased HF readmission risk (OR = 3.06, 95% CI: 1.59–5.88; *p* = 0.001). In contrast, among patients with normal BMI, no significant association was observed between the lowest TyG index (Q1) and the risk of HF readmission (OR = 0.80, 95% CI: 0.45–1.45; *p* = 0.470).

**Table 2:** Relationship between TyG index and readmission in HF patients with normal/abnormal BMI (model 3).

BMI category	Variable	Total	Event%	Crude OR (95% CI)	Crude *P* value	Adjust OR (95% CI)	Adjust *P* value
Normal BMI	Q1	121	43 (35.5)	0.92 (0.54∼1.55)	0.752	0.80 (0.45∼1.45)	0.470
	Q2	120	45 (37.5)	1 (Reference)		1 (Reference)	
	Q3	120	30 (25)	0.56 (0.32∼0.97)	0.038*	0.59 (0.32∼1.11)	0.101
	Q4	121	37 (30.6)	0.73 (0.43∼1.25)	0.257	0.64 (0.33∼1.24)	0.184
Abnormal BMI	Q1	105	49 (46.7)	2.76 (1.53∼4.99)	0.001*	3.06 (1.59∼5.88)	0.001*
	Q2	104	25 (24)	1 (Reference)		1 (Reference)	
	Q3	105	43 (41)	2.19 (1.21∼3.97)	0.01*	2.29 (1.18∼4.45)	0.014*
	Q4	105	38 (36.2)	1.79 (0.98∼3.27)	0.057	1.72 (0.82∼3.59)	0.152

TyG, triglyceride-glucose; HF, heart failure; BMI, body mass index; OR, Odds Ratio.

*P < 0.05.

### Incremental predictive value of the TyG index for HF readmission

**Figure 4:**
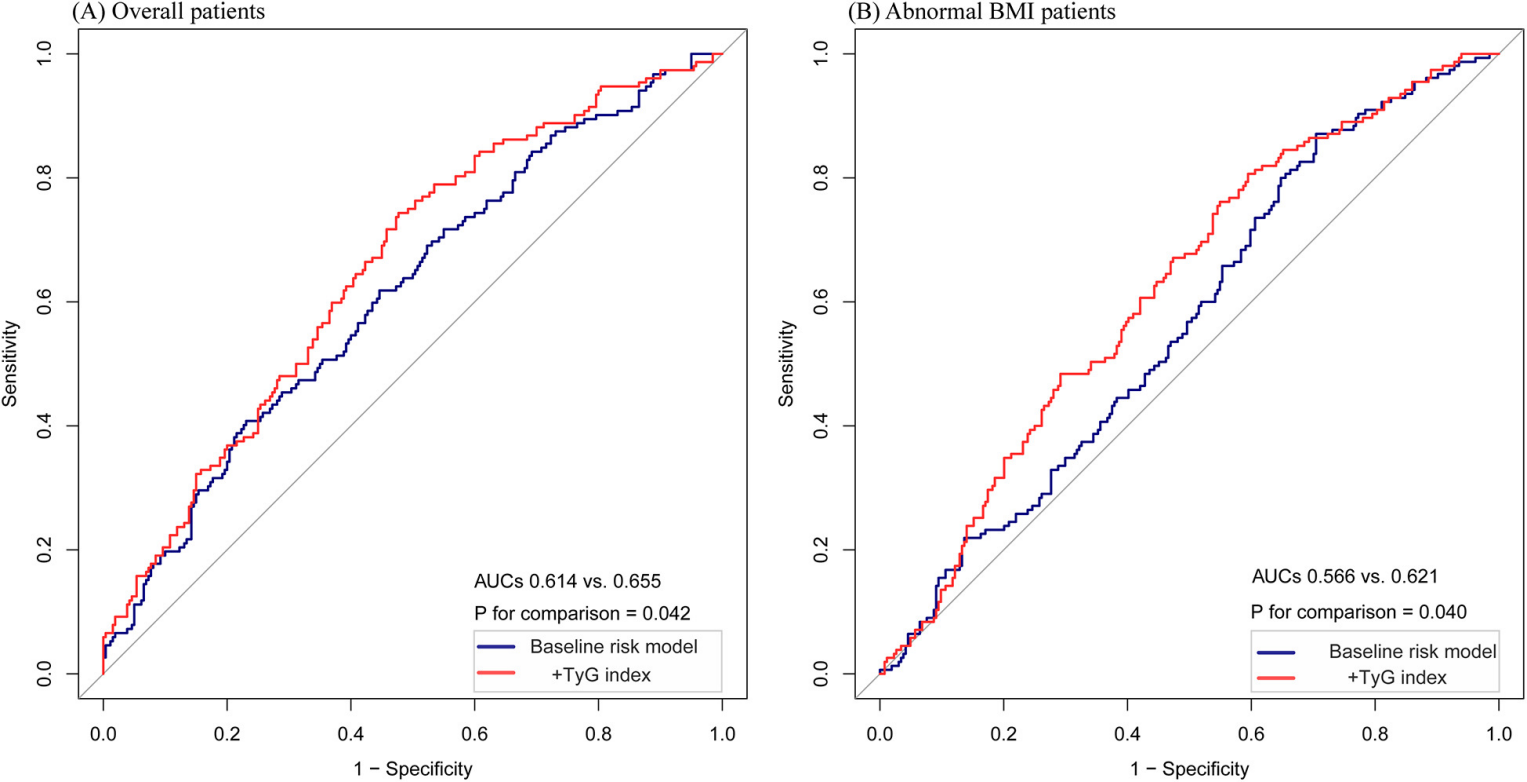
The ROC curves of the TyG index as a marker to predict HF readmission. **(A)** Baseline risk model vs.+TyG index in the overall patients. Baseline risk model includes diabetes, eGFR, calcium, albumin, cholesterol, and beta-blockers. **(B)** Baseline risk model vs.+TyG index in the abnormal BMI patients. Baseline risk model includes diabetes, calcium, albumin, hemoglobin, and statins. * *P* < 0.05.

In the overall patient population, ROC curve analysis was conducted for the baseline risk model, which included traditional risk factors such as diabetes, eGFR, calcium, albumin, cholesterol, and beta-blockers, along with a model enhanced by incorporating the TyG index as a categorical variable (Figure 4A). The inclusion of the TyG index led to a modest but statistically significant improvement in the AUC, increasing from 0.614 to 0.655 (*p* = 0.042). Furthermore, the category-free NRI of 0.0413 (*p* = 0.1866) and IDI of 0.0101 (*p* = 0.0042) (Table 3) provided evidence of a limited incremental contribution of the TyG index to the baseline risk model.

**Table 3:** NRI/IDI for models in all and abnormal BMI patients.

Group	Model comparison	Category-free NRI		IDI	
		Estimate (95% CI)	*P* value	Estimate (95% CI)	*P* value
Overall patients	Baseline risk model	1 (Reference)		1 (Reference)	
	+TyG Index	0.0413 (−0.0200 to 0.1025)	0.1866	0.0101 (0.0032–0.0170)	0.0042*
Abnormal BMI patients	Baseline risk model	1 (Reference)		1 (Reference)	
	+TyG index	0.0966 (−0.0173 to 0.2105)	0.0966	0.0277 (0.0120–0.0433)	0.0005*

(A) Baseline risk model vs.+TyG index in the overall patients. Baseline risk model includes diabetes, eGFR, calcium, albumin, cholesterol, and beta-blockers. (B) Baseline risk model vs.+TyG index in the abnormal BMI patients. Baseline risk model includes diabetes, calcium, albumin, hemoglobin, and statins. TyG, triglyceride-glucose; BMI, body mass index; eGFR, estimated glomerular filtration rate; NRI, net reclassification improvement; IDI, integrated discrimination improvement.

*P < 0.05.

In the subgroup of patients with abnormal BMI, ROC curve analysis was conducted for both the baseline risk model and the model incorporating the TyG index as a categorical variable (Figure 4B). In this subgroup, the improvement in AUC was more pronounced, increasing from 0.566 to 0.621 (*p* = 0.040). Moreover, the incorporation of the TyG index improved predictive performance metrics, as shown by the category-free NRI of 0.0966 (*p* = 0.0966) and IDI of 0.0277 (*p* = 0.0005) (Table 3). These findings suggest a greater incremental predictive significance of the TyG index for HF readmission in the subgroup of abnormal BMI patients.

## Discussion

This study identified a nonlinear relationship between the TyG index and the risk of 6-month readmission in HF patients. Lower TyG levels were correlated with an increased likelihood of readmission, particularly in patients with abnormal BMI. Incorporating the TyG index into the baseline risk model significantly enhanced its capability, especially in the abnormal BMI subgroup. These findings offer new perspectives on the possible function of the TyG index in HF risk stratification.

HF is considered the terminal phase of numerous cardiovascular illnesses. Despite considerable advancements in therapy and predictive tools, the incidence continues to increase (13). Therefore, identifying specific predictors of future HF events is vital for cardiovascular research. In clinical settings, various biomarkers, including type 2 diabetes mellitus (T2DM) (14) and IR (15), are currently used. Numerous studies have highlighted the strong relationship between IR and the onset and progression of HF (16). The TyG index is acknowledged as an affordable and dependable indicator for assessing IR (17), and clinical research has associated it with various diseases, including HF (18), coronary artery disease (19), hypertension (20), and stroke (21). Huang et al. have recognized the TyG index as an indicator of cardiovascular mortality and significant adverse cardiovascular events, and as a method for risk assessment in patients with acute decompensated HF (22). Similarly, Li et al. have shown that the TyG index independently influences the risk of HF (23). In this study, patients in the lowest TyG quartile (Q1) faced a notably greater risk of 6-month readmission compared to those in the second quartile (Q2), consistent across all adjusted models. Subgroup analyses revealed that this association was stronger in patients with abnormal BMI, where the lowest TyG quartile (Q1) was linked to increased readmission risk. These results indicate that the TyG index may serve as an important marker for detecting high-risk HF patients, particularly those with abnormal BMI.

Research utilizing data from the northern Chinese population has demonstrated that an increased TyG index is linked to a greater risk of HF onset (24). In the American demographic (25), the TyG index exhibited a nonlinear relationship with the duration of hospital stay among patients with HF. A retrospective cohort study involving a Chinese population identified a J-shaped dose-response relationship linking the TyG index to HF risk (26). Additionally, evidence suggests a U-shaped relationship, indicating that both lower and higher TyG indices are associated with poorer outcomes in HF patients (27). Consistent with previous findings, our study used smooth curve fitting and restricted cubic splines to uncover a nonlinear relationship between the TyG index and HF readmission, along with a connection between lower TyG index values and a higher likelihood of 6-month readmission. Elevated TyG index levels have traditionally been linked to worse cardiovascular outcomes, as they reliably indicate the degree of IR. However, our results suggest that lower TyG index levels are linked to a higher probability of HF readmission. This nonlinear pattern suggests that the TyG index, evaluated as a linear predictor, may not adequately reflect its complex relationship with HF prognosis. Then, a nonlinear relationship observed in this study indicates that the lower TyG levels may reflect underlying metabolic disturbances, potentially explaining the increased readmission risk in individuals exhibiting lower TyG index levels.

BMI, which is an indicator of IR (28), is also associated with HF prevalence (29). Both overweight and underweight individuals are at a higher risk of HF-related readmission (30, 31). Furthermore, studies have shown that the TyG index influences the relationship between BMI and the occurrence of HF (32). In a prospective study, Zheng et al. (33). have identified an interaction among BMI, TyG index, and HF incidence, revealing that increased TyG levels resulted in a greater likelihood of HF in individuals with normal BMI. Our study revealed that BMI and TyG index interact with the likelihood of HF rehospitalization. Specifically, within the abnormal BMI subgroup, a lower TyG index (Q1) was linked to an increased likelihood of HF rehospitalization. This association was further validated within the abnormal BMI population, which included overweight (21.9%) and underweight (24.6%) individuals. Evidence from prior studies indicates that IR levels are higher in these groups compared to individuals with normal BMI (32, 34). Notably, at the same lower TyG index level, patients with abnormal BMI had a higher risk of HF readmission, driven by elevated IR levels. These findings further indicates that IR may have an important influence on the likelihood of HF readmission.

Based on these findings, incorporating the TyG index into the baseline risk model greatly improved its predictive performance in the overall population, with especially notable improvements observed in patients with abnormal BMI. These improvements were supported by an increase in key metrics such as AUC and IDI, although the AUC of the predictive model remained modest, below 0.7, indicating limited overall predictive ability. This limitation may be partially attributed to specific characteristics of the study population, which consisted predominantly of elderly individuals (with 76% aged 70 years or older) and a high prevalence of multiple traditional cardiovascular risk factors. These factors likely added to the complexity of cardiovascular risk stratification, ultimately diminishing the model's discriminative capacity.

The mechanisms connecting the TyG index to readmission risk among patients with HF remain incompletely understood, with IR possibly playing a role. First, IR is related with a heightened likelihood of chronic metabolic conditions, including hypertension, diabetes, and dyslipidaemia, all of which are established risk factors for HF. Second, IR may cause lipotoxicity, leading to the release of inflammatory factors, impairment of nitric oxide function, and activation of both the sympathetic nervous and renin-angiotensin-aldosterone systems, resulting in cardiac dysfunction and myocardial injury (35). Third, our findings suggest a nonlinear relationship among the TyG index and HF readmission. Lower TyG index levels may trigger sudden physiological reactions, while moderate increases could activate compensatory mechanisms, reducing HF prevalence. However, excessive elevation of TyG levels may progressively impair the body's ability to compensate for IR, thereby heightening the risk of HF. However, further research is required to elucidate these mechanisms.

In spite of these results, this research has various limitations. First, the single-center observational study design did not allow definitive conclusions regarding causality. Second, we were unable to compare the TyG index with other current measures of IR owing to database limitations. Third, triglycerides, glucose, and other relevant parameters were measured only at baseline, and these might have changed during follow-up due to lifestyle changes and medications. Finally, the study population consisted primarily of elderly individuals with multiple comorbidities, which may have influenced the overall predictive performance of the model. Future studies should validate these results in larger, multi-center cohorts and explore the combined use of the TyG index with other biomarkers to improve HF risk stratification.

## Conclusion

A nonlinear relationship between the TyG index and 6-month readmission risk in HF patients. Subgroup analyses revealed that a lower TyG index was significantly connected to a higher readmission risk, especially in patients with abnormal BMI. Although the TyG index improved predictive performance, its overall discriminative ability remained modest, demonstrating greater utility in populations with abnormal BMI.

## Data Availability

Publicly available datasets were analyzed in this study. This data can be found here: https://doi.org/10.13026/8a9e-w734.
