# Supplementary material for: Association between triglyceride-glucose index and 6-month readmission in patients with heart failure: a cohort study
Source: Front Cardiovasc Med. 2025 Jul 29;12:1547755. doi: 10.3389/fcvm.2025.1547755 (PMC12339533; doi:10.3389/fcvm.2025.1547755)
Supplement: Supplementary file 1 [file Table1.pdf]

Supplementary table 1. Baseline characteristics of the patients

		TyG index				
		Q1(<8.20)	Q2(8.20~8.54)	Q3(8.54~8.96)	Q4(>8.96)	
Variables	Total (n = 901)	1 (n = 224)	2 (n = 226)	3 (n = 225)	4 (n = 226)	*P*
Age, n (%)						0.472
＜70	216 (24.0)	50 (22.3)	57 (25.2)	48 (21.3)	61 (27)	
≥70	685 (76.0)	174 (77.7)	169 (74.8)	177 (78.7)	165 (73)	
Gender, n (%)						< 0.001*
Male	401 (44.5)	108 (48.2)	115 (50.9)	103 (45.8)	75 (33.2)	
Female	500 (55.5)	116 (51.8)	111 (49.1)	122 (54.2)	151 (66.8)	
BMI (kg/m^2)						0.047*
normal	482 (53.5)	125 (55.8)	128 (56.6)	113 (50.2)	116 (51.3)	
abnormal	419 (46.5)	99 (44.2)	98 (43.4)	112 (49.8)	110 (48.7)	
≥24	197 (21.9)	35 (15.6)	46 (20.4)	53 (23.6)	63 (27.9)	
<18.5	222 (24.6)	64 (28.6)	52 (23)	59 (26.2)	47 (20.8)	
NYHA, n (%)						0.992
II	145 (16.1)	37 (16.4)	37 (16.5)	35 (15.6)	36 (15.9)	
III/ IV	756 (83.9)	189 (83.6)	187 (83.5)	190 (84.4)	190 (84.1)	
CHF, n (%)	838 (93.0)	206 (92)	216 (95.6)	206 (91.6)	210 (92.9)	0.334
PAD, n (%)	55 (6.1)	11 (4.9)	21 (9.3)	12 (5.3)	11 (4.9)	0.145
Cerebrovascular disease, n (%)	75 (8.3)	22 (9.8)	17 (7.5)	19 (8.4)	17 (7.5)	0.791
COPD, n (%)	143 (15.9)	42 (18.8)	44 (19.5)	26 (11.6)	31 (13.7)	0.057
Diabetes, n (%)	232 (25.7)	29 (12.9)	41 (18.1)	49 (21.8)	113 (50)	< 0.001*
CKD, n (%)	219 (24.3)	48 (21.4)	55 (24.3)	48 (21.3)	68 (30.1)	0.102
6-month readmission, n (%)	310 (34.4)	94 (42)	67 (29.6)	74 (32.9)	75 (33.2)	0.04*
TyG index	8.6 ± 0.7	7.9 ± 0.3	8.4 ± 0.1	8.7 ± 0.1	9.6 ± 0.6	< 0.001*
White blood cell (10^9/L)	7.6 ± 3.8	6.4 ± 3.9	7.0 ± 2.7	7.8 ± 3.2	9.3 ± 4.4	< 0.001*
Hemoglobin (g/L)	115.7 ± 24.3	110.9 ± 24.5	114.9 ± 23.3	119.1 ± 24.2	117.9 ± 24.7	0.002*
Platelet (10^9/L)	146.2 ± 64.1	125.9 ± 56.5	144.4 ± 67.1	153.0 ± 61.1	161.6 ± 66.2	< 0.001*
eGFR（mL/min/1.73 m^2）	69.3 ± 37.1	76.4 ± 41.8	68.8 ± 36.1	66.6 ± 31.6	65.6 ± 37.5	0.009*
Calcium (mmol/L)	2.3 ± 0.2	2.2 ± 0.2	2.3 ± 0.2	2.3 ± 0.2	2.3 ± 0.2	0.005*
Lactate (mmol/L)	2.3 ± 1.7	2.1 ± 2.1	2.2 ± 1.4	2.2 ± 1.1	2.5 ± 1.8	0.032*

BNP (pg/mL)	1383.4 ± 1435.2	1255.1 ± 1313.1	1532.8 ± 1497.0	1506.0 ± 1525.3	1240.0 ± 1379.2	0.044*
Albumin (g/L)	36.2 ± 4.9	35.3 ± 4.6	35.9 ± 4.7	36.6 ± 4.7	36.9 ± 5.3	0.003*
Cholesterol (mmol/L)	3.8 ± 1.1	3.2 ± 0.9	3.6 ± 0.9	3.9 ± 1.0	4.4 ± 1.4	< 0.001*
Vasodilators, n (%)	244 (27.1)	45 (20.1)	54 (23.9)	66 (29.3)	79 (35)	0.002*
Beta-blockers, n (%)	338 (37.5)	71 (31.7)	73 (32.3)	89 (39.6)	105 (46.5)	0.003*
RAS inhibitors, n (%)	308 (34.2)	66 (29.5)	78 (34.5)	86 (38.2)	78 (34.5)	0.276
Inotropes , n (%)	791 (87.8)	198 (88.4)	202 (89.4)	197 (87.6)	194 (85.8)	0.7
Diuretics , n (%)	891 (98.9)	224 (100)	223 (98.7)	223 (99.1)	221 (97.8)	0.169
Statins, n (%)	396 (44.0)	85 (37.9)	93 (41.2)	98 (43.6)	120 (53.1)	0.009

Data are presented as mean ± SD, median (Interquartile range) or n (%).TyG, triglyceride-glucose; HF, heart failure; BMI, body mass index; NYHA, New York Heart Association; CHF, congestive heart failure; PAD, peripheral vascular disease; COPD, chronic obstructive pulmonary disease; CKD, chronic kidney disease; eGFR, estimated glomerular filtration rate; BNP, brain natriuretic peptide; LDL-C, low-density lipoprotein cholesterol; HDL-C, high-density lipoprotein cholesterol; RAS inhibitors, renin-angiotensin-aldosterone system inhibitors.* $P < 0.05$.
